# Why can't we be friends? Exploring factors associated with cat owners' perceptions of the cat-cat relationship in two-cat households

**DOI:** 10.3389/fvets.2023.1128757

**Published:** 2023-03-27

**Authors:** Sherry Khoddami, Makayla C. Kiser, Carly M. Moody

**Affiliations:** ^1^Faculty of Land and Food Systems, University of British Columbia, Vancouver, BC, Canada; ^2^Department of Animal Science, University of California, Davis, Davis, CA, United States

**Keywords:** multi-cat household, inter-cat conflict, cat behavior, cat welfare, social structure

## Abstract

Most research examining cat behavior in multi-cat households lacks focus on one group size. This gap in knowledge reduces generalizability of research findings to specific compositions of cats in multi-cat households. Given that many cat-owning households in Canada and the US are comprised of two cats, the following study used a cross-sectional survey to explore cat owners' perceptions of the cat-cat relationship in two-cat households in Canada and the US. A total of 6,529 owners of two cats completed the online questionnaire. Descriptive statistics were used to summarize the data and a logistic regression model used to assess various explanatory variables (i.e., household, management, and cat-specific factors) associated with participants perceiving their cats' relationship as negative. The logistic regression model showed that owners of two-cat households are more likely to perceive their cats' relationship as negative if both cats are spayed females, adult or mature, have a large gap in age, not related, one or both have access to the outdoors, or show aggression toward people or other animals in the home. Having multiple litterbox and feeding areas were also associated with a more negative cat-cat relationship. Overall, the complex interplay, directionality, and temporality of these factors requires further investigation for a full understanding of how to improve the cat-cat relationship in two-cat households. More research is needed to provide evidence-based recommendations for managing and supporting a positive cat-cat relationship in the home.

## 1. Introduction

In Canada and the US there are an estimated 70 million pet cats (1, 2), with many cat-owning households consisting of two cats [average of 1.6 cats/ Canadian household, average of 1.8 cats/ US household; (1, 2)]. Although current house cats descend from a wildcat thought to be largely solitary (*Felis silvestris lybica*), partial domestication has adapted cats to group living associated with food availability near human settlements (3). As cats made their way into our homes, owners often choose to house cats with conspecifics, without understanding the impact on the cats themselves. Thus, multi-cat households vary in composition, with cats housed together that are unrelated, related, acquired together, and/or acquired separately. The welfare of pet cats in multi-cat households is dependent on many factors including their relationship with conspecifics (4, 5), the physical home environment (6–8), and caretaker interactions (4, 9). Cat-cat interactions include positively valenced interactions such as affiliative behaviors (example: allo-grooming, playing), as well as negatively valenced interactions such as agonism (example: staring, resource guarding), with negative interactions garnering more research and attention.

Inter-cat conflict is one of the most frequent owner-reported problems in multi-cat households (4, 10–12) and one of the main reasons cats are presented to behavior clinics (13–16). A UK-based survey suggests 62% (*N* = 616 cats) of multi-cat owners see signs of inter-cat conflict (hissing, spitting, or blocking) in their household (17). Similarly, 50% of cat-owner participants from a Canadian survey assessing (*N* = 1,146) fostered kittens adopted into multi-cat households, reported seeing behavioral signs of aggression between cats in their home (18). When inter-cat conflict is not mitigated, the welfare of both cats may be compromised due to prolonged stress (5, 19) leading to health and behavioral problems such as house soiling (20–22), as well as increased risk of owner-directed bites and scratches (23). In addition, behavior problems can diminish the human-animal bond (9, 24) and may lead to relinquishment (25–27).

Many factors impact the cat-cat relationship, including resource provision, outdoor access, and cat characteristics (28–30). Research shows that inadequate resource availability and distribution may increase inter-cat conflict in the home (30). Thus, it is recommended to provide multiple, well-distributed resources (example: food bowls, litter boxes, and environmental enrichment items) throughout the home to reduce individuals from monopolizing resources (30). To further support this recommendation, observational studies of group-housed indoor cats have found that cats time-share resources, meaning they choose to access valuable resources at different times (29, 31). A large survey of US and Canadian cat owners by Tan and colleagues shows that owners providing cats with uncontrolled outdoor access are less likely to provide important in-home resources such as those necessary for cats to perform naturally motivated behaviors such as perching and playing (32). In addition, allowing cats outdoors has been associated with continued fighting (12) and an increased likelihood of aggression between household cats (18). However, in contrast, other research has not found associations between outdoor access and agonistic interactions in multi-cat households (33, 34). Although no consensus exists, the study by Tan and colleagues suggests that uncontrolled outdoor access is more likely to be provided by owners of cats showing aggression toward other cats or people in the home (32). Thus, there is some evidence to support a relationship exists, however the temporal directionality of this relationship is unknown.

Cat characteristics such as sex, age, weaning age, neuter status, as well as familiarity and relatedness have been suggested to impact the cat relationship in multi-cat households. Neuter status is known to reduce conflict behavior in both neutered male and female cats compared to intact cats of the same sex (35). Barry and Crowell-Davis (36) found that indoor, neutered males choose to spend more time in close proximity than other sex combinations. As well, age may impact cat-cat behavior; for example, Ramos and colleagues (37) found adult cats have significantly higher fecal glucocorticoid levels than young cats in multi-cat households (3-4 cats). As well, a UK-based survey of cat owners (*N* = 833) suggests cats 11 years and older tend to be less sociable toward other animals (including dogs, cats, etc.) in the home as they get older (38). In contrast, one survey (*N* = 2492) reports that young and adult cats display more active conflict-related behaviors (such as chasing and stalking) than households with mature or senior cats (34). However, chasing and stalking are also indicative of play behavior in cats, and thus may not be negative for all cats involved. As cats age, they are at an increased risk of developing diseases which may cause pain or discomfort (39), and thus their tolerance of interactions with other cats may change. The age at which cats are weaned from their mother also impacts the inter-cat relationship, with early weaned cats (< 8 weeks of age) more likely to show aggression toward other cats (40). Furthermore, kittens that are reared without their littermates during the early socialization period (approximately between 2 and 7 weeks old) display more agonistic interactions with conspecifics (41). Generally, social deprivation from the mother and littermates during the socialization period may negatively influence cats' subsequent social interactions with conspecifics (4). In addition, related and unrelated but familiar kittens and cats show more affiliative interactions compared to unfamiliar and unrelated cats (42–44).

Although there is an increasing body of research examining factors impacting the inter-cat relationship in multi-cat households, one large problem is the lack of research focusing on one group size. This gap in knowledge reduces generalizability of the research findings to specific compositions of cats in multi-cat households (10). This is important, given that it is generally recognized that varying the number of animals in a group impacts social structure and social complexity (45). Studies assessing multi-cat households often include large cohorts of cats (17, 29, 34, 46) which may have an increased risk of conflict compared to smaller cohorts of cats (34). To the authors' knowledge, one study has examined indoor-only cat dyads in the home, with results suggesting that neutered male dyads spend more time in close proximity than female dyads or male-female dyads (36). However, more research is needed to assess these findings in a larger population and achieve a better understanding of the factors impacting cat dyad behavior in the home. Overall, research focused on improved understanding of the social relationship between cats of a defined number in a household is needed. Since many households in Canada and the US contain two cats (1, 2), research focused on two-cat households may be more impactful. Given the limited research focusing on two-cat households, we aimed to explore and describe associations between cat owners' perceptions of their cats' relationship, management factors, and cat-specific characteristics. A cross-sectional questionnaire was used to survey US and Canadian owners of two adult cats (≥1 year old) and included questions on owner demographics, owner's self-perceived knowledge of cat behavior, owner's subjective perception of their cats overall relationship, household and management factors, and cat dyad characteristics. We predicted that cat owners rating their cats as having a negative relationship would be associated with provision of a single resource area (example: one litter box, one feeding area) compared to households with multiple resource areas (example: two or more litter boxes and feeding areas), and reduced odds of a negative relationship in households with related cats compared to unrelated cats.

## 2. Materials and methods

This study was approved by the University of California Davis Institutional Review Board (IRB #1786341-1) to recruit human participants for research. Participation was anonymous and respondents provided consent before being able to participate in the questionnaire.

### 2.1. Data collection

An online cross-sectional questionnaire was developed using an online survey software program (Qualtrics Software Company, Provo, Utah, USA). Participation required respondents to be at least 18 years old, currently living in Canada or the USA, and identify as the current primary owner of two companion cats that spend at least fifty percent of their time indoors. The survey was in English and required internet access to participate. Recruitment involved advertising on social media sites such as Facebook and Twitter using snowball sampling (47). Data collection occurred during September 13th–17th, 2021.

### 2.2. Questionnaire

The questionnaire consisted of five sections and 77 questions total: (1) inclusion criteria (4 questions), (2) participant demographics (nine questions), (3) resource provision and distribution (four questions), (4) cat characteristics, health and behavior information, and cat-cat interactions (20 questions), and (5) 10 videos depicting various two-cat interactions asking participants to rate each video's cat-cat interaction, and rate how often they see their own cats display similar behaviors (40 questions). The research reported here includes questionnaire sections 1–4; section 5 is not reported here.

Participant demographic questions included age (18–29, 30–39, 40–49, 50–59, 60–69, 70+, prefer not to say but over 18), gender (male, female, non-binary, other, prefer not to say), US state or Canadian province currently residing, self-perceived knowledge level of cat behavior (extremely, very, moderately, somewhat, not at all), previous experience working with companion cats (yes, no) and if yes, number of years of combined experience (1 year, 1–5 years, 6–10 years, 11–15 years, 16+ years). We also asked questions designed to understand the cats' household including the area of the household (< 500 square feet (sq ft), 500–1,000 sq ft, 1,000–1,500 sq ft, >1,500 sq ft, prefer not to answer), the total number of adults (18 years of age or older; numeric entry), children (< 18 years of age; numeric entry), and dogs in the household (0, 1, 2, 3, 4+). Cat specific information asked about each cat including their names, declaw status (no, yes all 4 paws, yes front paws only, yes back paws only, not sure), where each were obtained (breeder, pet store, shelter or rescue, family friend relative or neighbor, found as stray or feral cat, previous cat's litter), breed (domestic, purebred, purebred mix, not sure), coat pattern (select all that apply: solid, tabby, bi-color, tortoiseshell, calico, other), coat color (select all that apply: beige, black, brown, gray, lavender, orange, red, white), sex (female spayed, male neutered, female intact, male intact, not sure), current age in years (numeric entry), age introduced into the home (< 1 year, 1–3 years old, 4–6 years old, 7–10 years old, >10 years old), outdoor access (strictly indoor, indoor with supervised outdoor access, indoor with unsupervised outdoor access), and current/previous health and behavioral problems (see Supplementary material for full questionnaire).

Questions about the owner's perspective of the cats' relationship asked participants to rate the valence of their cats' first encounter, as well as their current overall relationship using a 5-point Likert scale (extremely negative, somewhat negative, neutral, somewhat positive, extremely positive, not sure or previously introduced). Other cat-cat relationship questions included relatedness (not related, siblings, mother and offspring, father and offspring, other) and time spent living together (< 1 year, 1–3 years, 4–6 years, 7–9 years, 10+ years).

Resource questions were designed to understand the number and distribution of resources in two-cat households. To reduce competition in multi-cat households, behaviorists and veterinarians recommend placing multiple resources (i.e., litter boxes, food, and water stations) in different locations, and suggest that two of the same resources in close proximity may be viewed as a single resource by cats (48). Based on this reasoning, if two litter boxes are side by side this should count as one litter box. It is also suggested that multi-cat households follow the *n* + *1 rule* for determining the number of litter boxes to provide, with “n” being the number of cats in the household (49). Thus, in a two-cat household, the gold standard would be to provide 3 litter boxes dispersed around the home. It should be noted there is no scientific evidence to corroborate these recommendations. Nonetheless, based on these recommendations, we asked participants how many scratching posts (0–10+), litter boxes, food bowls, and sleeping areas (in the same room side by side, in the same room not side by side, in different rooms, one resource is provided, no resource is provided) they provide in their household. Since cats may sleep on various surfaces and areas throughout the home, the following examples were provided: cat beds, owners' bed, furniture, and cat trees or hammocks. Perching and hiding areas were not included in the questionnaire, despite their importance for cat welfare (5, 7, 50). Since different areas in the home may be used for perching (example: cat tree or shelves) and hiding (ex. behind furniture, under bed) owners may have difficulty identifying and quantifying these areas which may reduce accuracy of the data.

### 2.3. Statistical analysis

Only complete responses were included in analyses, and thus incomplete and duplicate responses from the same IP address were excluded. Data from 6,529 owners of two cats (*N* = 13,058 cats) were included for analysis. To reduce misclassification bias during data cleaning, questions with the option ‘other' and participant typed responses were evaluated to ensure accurate response allocation. Descriptive statistics (percentages, frequencies) were generated using RStudio (Auckland, New Zealand), and all other analyses were conducted using SAS Studio v3.7 (SAS Institute, Cary, North Carolina, USA). Descriptive statistics were generated for each survey question, initially by country (US and Canada) and later combined due to their similarity.

A logistic regression model was used to evaluate explanatory variables associated with participants rating their cats' relationship as negative. The Likert-scale variable “overall relationship” was consolidated to create a binary outcome variable: extremely positive and somewhat positive were combined into a “positive” category, while somewhat negative and extremely negative were combined into a “negative” category; neutral (*n* = 895) was not included as it did not fit into a binary positive/negative variable). Potential explanatory variables included a total of 48 variables (cat owner demographics, cat characteristics and relationship information, resource variables, and health and behavior variables), and thus many were collapsed to simplify the variables for analyses. For example, the 16 health variables (i.e., diabetes, osteoarthritis, heart disease) were combined to create ‘at least one cat in household with a health condition (yes/no)' variable to assess the overall impact of health conditions on the cat-cat relationship. Similarly, an overall “at least one cat in household with a behavior problem (yes/no)” variable was created by collapsing the 10 behavior problem variables (i.e., animal-directed aggression, human-directed aggression, excessive night time activity). However, individual behavior problem and health condition variables were also tested for inclusion in the model. Other explanatory variables tested for inclusion in the model were: owner and household variables (household size, number of adults in the house, dogs in the house, children in the house, owner's knowledge of cat behavior, owner's experience working with cats), cat demographics (both cats' sex, breed combinations, cats' age combinations, ages of cats when obtained, where cats were obtained, cats' relatedness, time living together, declaw status combinations, and outdoor access), and resource-related information (feeding areas, litter box areas, sleeping areas, and number of scratching posts). First encounter data were not included in the model as we found misclassification bias present in this variable. Of the respondents, 25.6% reported their cats to be related or previously introduced, answered the first encounter question, even though their cats' would not require an introduction. Given this, we did not analyze this variable any further.

To evaluate which of the variables should be included in the model, two-way analyses were run with each potential explanatory variable and the outcome (overall cat-cat relationship). A liberal *p*-value (*p* < 0.2) was used to guide which variables to include in the model. The final logistic regression model was built using a stepwise model building strategy where variables with a *p* < 0.05 were retained. All plausible two-way interactions were testing during model building, and due to all explanatory variables being categorical, model fit was based on evaluation of the 2-way interaction terms. *Post-hoc* pairwise comparisons with 4 or more pairs used a Tukey's adjustment for multiple comparisons to reduce the potential for type I errors. Results are reported using odds ratios (OR), 95% CI's and *p*-values.

## 3. Results

### 3.1. Descriptive results

The majority of survey participants (*N* = 6,529) resided in the US (6,118/6,529, 93.7%; Table 1), with the most frequently reported states being California (15.5%), New York (6.3%), Texas (5.5%), and Washington (5%). Of Canadian respondents (411/6,529, 6.3%), the provinces most frequently reported was Ontario (45.5%), British Columbia (22.9%), Alberta (10.9%), Quebec (7.3%), and Nova Scotia (5.1%). In total, the majority of respondents were female (71.5%) and living in a household with two adults including the participant (63.1%; Table 2), live with no dogs (76.4%) and no children (77.8%). The most frequently selected household area was more than 1,500 square feet (41.1%) and most frequently selected age ranges were 30–39 (31.4%) and 40–49 (24.4%) years old. Most participants indicated they did not have work experience with cats (72.4%) and most frequently rated themselves on a Likert scale as “very knowledgeable” about cat behavior (44.5%). Of the participants with cat-related work experience (27.6%, 1,802/6,529), 40.7% had more than 16 years of experience.

**Table 1:** Demographic descriptive information for 6,529 owners of two adult cats residing in US or Canada that completed the online questionnaire regarding resource provision and perception of their cats' overall relationship.

**Variable**	**Category**	**No. (%) of respondents**
Country	USA	6,118 (93.7)
	Canada	411 (6.3)
Age	30–39	2,053 (31.4)
	40–49	1,590 (24.4)
	50–59	1,108 (17.0)
	18–29	758 (11.6)
	60–69	739 (11.3)
	70+	229 (3.5)
	Prefer not to say	52 (0.8)
Gender	Female	4,669 (71.5)
	Male	1,635 (25.0)
	Non-binary	151 (2.3)
	Prefer not to say	61 (0.9)
	Other	13 (0.2)
Previous cat experience	No	4,725 (72.4)
	Yes	1,804 (27.6)
Years of experience (if “Yes” to above)	16+	734 (40.7)
	1–5	444 (24.6)
	6–10	283 (15.7)
	11–15	184 (10.2)
	< 1	159 (8.8)
Self-reported knowledge of cat behavior	Very knowledgeable	2,908 (44.5)
	Moderately knowledgeable	2,211 (33.9)
	Extremely knowledgeable	1,036 (15.9)
	Somewhat knowledgeable	364 (5.6)
	Not at all knowledgeable	10 (0.2)

**Table 2:** Household descriptive information for 6,529 owners of two adult cats residing in US or Canada that completed the online questionnaire regarding resource provision and perception of their cats' overall relationship.

**Variable**	**Category**	**No. (%) of respondents**
Household area	More than 1,500 sq ft	2,683 (41.1)
	1,000–1,500 sq ft	2,108 (32.2)
	500–1,000 sq ft	1,544 (23.6)
	< 500 sq ft	1,33 (2.0)
	Prefer not to say	61 (0.9)
Dogs	0	4,988 (76.4)
	1	948 (14.5)
	2	440 (6.7)
	3+	153 (2.4)
Adults	2	4,116 (63.1)
	1	1,612 (24.7)
	3	557 (8.5)
	4+	178 (2.7)
	0	64 (1.0)
Children	0	5,076 (77.8)
	1	749 (11.5)
	2	540 (8.3)
	3+	163 (2.5)

A few participants did not answer all questions, therefore the number of responses varies among variables.

Response data from 13,058 cats were analyzed (two cats per respondent). Respondents' cats were 49.8% neutered males and 49.3% spayed females (Table 3). Most were not declawed (92.1%) and were acquired from a shelter (59.7%). Cats were most frequently acquired at kitten age (73.8%), 1−3 years old (30.6%), domestic breed (76.4%), and had a tabby coat pattern (35.5%). Most participants indicated that their cats are strictly indoors-only (67.1%) and some reported providing supervised outdoor access (23.5%).

**Table 3:** Cat descriptive information collected from 6,529 US and Canadian owners of 2 cats (total of 13,058 cats) who completed an online questionnaire.

**Variable**	**Category**	**No. (%) of respondents**
Sex	Male neutered	6,508 (49.8)
	Female spayed	6,439 (49.3)
	Female intact	77 (0.6)
	Male intact	30 (0.2)
	Not sure	4 (0)
Declaw status	No	12,020 (92.1)
	Yes, all four paws	120 (0.9)
	Yes, only front paws	915 (7.0)
	Yes, only back paws	3 (0.0)
Origin	Shelter	7,794 (59.7)
	Family or friends	2,165 (16.6)
	Found	2,148 (16.4)
	Other	951 (7.3)
Age adopted	Kitten (0–1 year)	9,641 (73.8)
	Young adult (1–3 years)	2,382 (18.2)
	Adult (4–6 years)	662 (5.1)
	Mature/senior (7+ years)	373 (2.9)
Age (in years)	1–3	3,971 (30.6)
	4–6	3,124 (24.1)
	7–10	3,122 (24)
	10+	2,768 (21.3)
Breed	Domestic	9,977 (76.4)
	Not sure	1,828 (14.0)
	Purebred	832 (6.4)
	Purebred mix	421 (3.2)
Outdoor access	Indoor only	8,758 (67.1)
	Indoor + supervised outdoor	3,073 (23.5)
	Indoor + unsupervised outdoor	1,227 (9.4)
Coat pattern	Tabby	4,630 (35.5)
	Bicolor	2,948 (22.6)
	Solid	2,541 (19.5)
	Other	880 (6.7)
	Tortoiseshell	755 (5.8)
	Calico	713 (5.5)
	Mixed patterns	591 (4.5)

Some participants did not answer all questions, therefore the number of responses varies among variables.

Participants most frequently rated (Likert scale: extremely positive, somewhat positive, neutral, somewhat negative, extremely negative) their cats' first encounter as “somewhat negative” (26.2%) or “extremely positive” (24.1%), and their cats' overall relationship as “extremely positive” (39.8%) or “somewhat positive” (33.7%; Table 4).

**Table 4:** Owner's perception of their cats' relationship collected from 6,529 owners of two cats in US and Canada.

**Variable**	**Category**	**No. (%) of respondents**
First encounter	Somewhat negative	1,713 (26.2)
	Extremely positive	1,575 (24.1)
	Neutral	1,145 (17.5)
	Somewhat positive	858 (13.1)
	Previously introduced	545 (8.3)
	Extremely negative	470 (7.2)
	Not sure	223 (3.4)
Overall relationship	Extremely positive	2,598 (39.8)
	Somewhat positive	2,199 (33.7)
	Neither positive nor negative	895 (13.7)
	Somewhat negative	747 (11.4)
	Extremely negative	90 (1.4)
Time together (years)	1–3	2,640 (40.4)
	4–6	1,582 (24.2)
	7–9	935 (14.3)
	10+	904 (13.8)
	< 1	468 (7.2)
Cats' relation	Not related	4,620 (70.8)
	Siblings	1,673 (25.6)
	Other	236 (3.6)

When asked about resource distribution in the home, most respondents indicated they provide their cats with a single feeding area (59.1%; multiple areas: 40.9%), single litter box area (57.1%; multiple litter box areas: 42.1%; no litterbox: 0.8%), and multiple sleeping areas (83.4%; single sleeping area: 10.0%). When asked about the quantity of scratching posts, respondents provided 4 or more posts (30.7%), 2 posts (26.1%), 3 posts (22.1%), 1 post (15.5%), or none (5.6%).

The majority of cat owners reported at least one cat (3,911/ 6,529, 59.9%) in their household has ≥ 1 current or previous diagnosed health issue (Table 5). Of cats with at least one current or previous health issue, the most frequently reported health issues were obesity (25.2%) and dental disease (23.4%). The majority of cat owners also reported that at least one cat (78.5%) in their household has ≥1 current or previous behavioral issue (Table 6). The most frequently reported behavioral issues were fears/phobias (45.7%), unwanted behaviors (45.2%) and destructive behaviors (40.6%). Almost half of participants reported at least one cat (49.7%) in their household has ≥1 current or previous health and behavioral issue.

**Table 5:** Current and/or previously diagnosed health issues reported for at least one cat by 6,529 US and Canadian owners of two cats (*N* = 13,058 cats).

**Health issues**	***N***	**%**
Obesity	984	25.2
Dental disease	915	23.4
Other	763	19.5
Gastrointestinal disorders	685	17.5
Dermatological disorders	659	16.9
Eye disorders	622	15.9
External parasites	611	15.6
Non-obstructive urinary diseases	388	9.9
Obstructive urinary diseases	364	9.3
Respiratory diseases	342	8.8
Internal parasites	318	8.1
Hypothyroidism	217	5.6
Renal disease	197	5
Osteoarthritis	153	3.9
Diabetes	140	3.6
Heart disease	136	3.5

**Table 6:** Current and/or previous behavioral issues reported for at least 1 cat by 6,529 US and Canadian owners of 2 cats (*N* = 13,058 cats).

**Behavioral issues**	***N***	**%**
Fear/phobias	2,346	45.7
Unwanted behaviors	2,317	45.2
Destructive behaviors	2,080	40.6
Separation anxiety	1,355	26.4
Animal aggression	1,179	23
Stereotypic and compulsive disorders	1,052	20.5
Excessive night time activity	789	15.4
People aggression	615	12
Gastrointestinal and ingestive disorders	575	11.2
Other	537	10.5

### 3.2. Logistic regression model results

Factors that influenced cat dyads having a negative relationship are presented in Table 7 with associated ORs, 95% CIs, and *p*-values. The final model included explanatory variables: outdoor access, sex, age, litter box areas, feeding areas, cat aggression shown toward people, cat aggression shown toward other animals, and relatedness. No other significant effects were detected.

**Table 7:** Multi-level logistic regression model results showing social and physical environmental factors associated with two cats from the same household having an overall more negative relationship, based on owner perception (*N* = 6,529 participants).

**Explanatory variables**	**Category**	**OR (95% CI)**	***P*-value**
Feeding areas	Single (Ref)	-	-
	Multiple	2.04 (1.72–2.42)	< 0.0001
Litterbox areas	Single (Ref)	-	-
	Multiple	1.48 (1.25–1.76)	< 0.0001
Sex	NM and SF (Ref)	-	-
	Both SF	1.61 (1.23–2.11)	^*^ < 0.0001
	Both NM (Ref)	-	-
	Both SF	3.32 (2.34–4.72)	^*^ < 0.0001
	NM and SF	2.07 (1.48–2.88)	^*^ < 0.0001
Age groups	Both young (Ref)	-	-
	Adult and mature	3.89 (2.32–6.53)	^*^ < 0.0001
	Both adult	2.46 (1.41–4.29)	^*^ < 0.0001
	Both mature	4.15 (2.63–6.54)	^*^ < 0.0001
	Young and adult	0.45 (0.26–0.81)	^*^0.0012
	Young and mature	0.28 (0.17–0.48)	^*^ < 0.0001
	Young and adult (Ref)	-	-
	Adult and mature	1.77 (1.09–2.86)	^*^0.0098
	Both mature	1.88 (1.22–2.90)	^*^0.0004
	Both adult (Ref)	-	-
	Both mature	0.59 (0.39–0.90)	^*^0.0049
Relation	Other (Ref)	-	-
	Not related	2.68 (1.50–4.80)	0.0009
	Siblings (Ref)	-	-
	Not related	2.02 (1.57–2.60)	< 0.0001
Aggression toward people	No (Ref)	-	-
	Yes	0.69 (0.54–0.88)	0.0025
Aggression toward animals	No (Ref)	-	-
	Yes	0.24 (0.20–0.29)	< 0.0001
Outdoor access	Both indoor (Ref)	-	-
	Both outdoor	0.72 (0.60–0.86)	0.0004
	One indoor, one outdoor	0.60 (0.46–0.78)	0.0002

^*^Tukey adjusted p-value and adjusted confidence interval used.

## 4. Discussion

Our survey results suggest many factors impact cat owner ratings of their cats' relationship. Interestingly, spayed female dyads were more likely to have an owner perceived negative relationship compared to neutered male dyads, or mixed sex dyads. This is in line with other literature on the influence of sex on the cat-cat relationship in the home. One study by Barry and Crowell-Davis (36) shows that indoor neutered male dyads choose to spend more time in closer proximity than females or mixed sex combinations, suggesting male dyads may get along better than other sex combinations. However, there is conflicting results from behavioral clinic data, with one study from Australia suggesting that female cats display more inter-cat aggression than male cats (13), while another study in the US by Lindell and colleagues (4) suggests that male cats are more likely to act as aggressors toward other male or female cats. Albeit, behavioral clinic data stems from a limited sample and likely represents more severe cases of inter-cat conflict that may not be generalizable to the average two-cat household. Given that our results, as well as much of the existing scientific evidence, suggests spayed female dyads show more negative interactions, this could be something for cat adopters to consider when they already have one female cat at home. It should be noted that other factors such as age of weaning, socialization experiences, and how cats are introduced, likely impact the complex relationship between cat dyads in the home. As well, animal shelters typically place cats into a home with another cat if the shelter cat has a history of living with other cats, and if they display more social behaviors in the shelter or foster home such as playing, compared to fearful or avoidant behaviors (51).

Age also impacted owner ratings of their cats' relationship, with younger (1–3 years old) cat dyads less likely to be rated negatively, compared to all other age group combinations. In addition, dyads consisting of a young and adult cat (4–6 years old) were less likely to have a negative relationship compared to mature-cat combinations. As well, pairs of mature cats (7+ years old) were more likely to have a negatively perceived relationship compared to pairs of adult cats. This suggests that cat owners perceive younger cats as getting along better with other young or adult cats, compared to mature cat combinations. Thus, when pairing cats, such as during the adoption process, it may be beneficial to pair younger cats together and avoid mature-cat combinations. Other research examining age-related impacts on the multi-cat relationship suggests that younger cats display chasing and fleeing behaviors, which the authors categorized as conflict-related, more frequently than older cats (34). However, chasing and fleeing behaviors are also seen during play (52), and thus may indicate increased play and not conflict, in younger vs. older cats. Another study examining the impact of conflict behaviors in households following the introduction of a new cat did not find an age effect on the cat-cat relationship (12). Overall, more research is needed to establish stronger links between cat dyad age combinations and the cat-cat relationship. For example, a prospective cohort study with direct behavioral observations of cat dyads of various age combinations in two-cat households would be beneficial.

Cats' relatedness was a factor that impacted participant ratings of their cats' relationship. Cats that were not related were more likely to be rated as having a negative relationship compared to cats that were siblings or placed into the “other” category. Participants that selected “other” had cats that were parent and child or that were bonded before adoption. This finding is not unexpected given the natural history of cats. For example, in free ranging cat colonies, individuals choose to socialize with preferred conspecifics and related females typically interact and may even form small colonies with other females from their lineage and their offspring (3, 11). Similarly in a private colony of neutered cats, Curtis and colleagues (42) found related cats were significantly more likely to be within 1 m of each other and display allogrooming, an affiliative inter-cat behavior. In shelter environments, littermates show more physical contact and allogrooming behavior than unrelated cats from the same household (43). Thus, related cats may be more likely to get along due to the strong bonds formed early in life (11, 43). It is important to educate owners on the importance of relatedness and early social bonds when they are adopting. If there is an opportunity to adopt related cats, owners should be educated on the positive influence it may have on the cats' relationship and the possible consequences of introducing an unrelated cat later on. Future studies should investigate the motivations of cat owners seeking additional cats into their households. Further, research is needed to provide evidence-based recommendations for introducing an unknown cat into a household with existing cat(s).

Outdoor access (either supervised or unsupervised), was another factor that impacted participant ratings of the cat-cat relationship in 2-cat households; those with outdoor access were more likely to have a reported negative relationship than pairs kept indoors-only. Other cross-sectional cat-owner survey research examining multi-cat households have found similar results, with outdoor access associated with increased aggression toward other household cats (18), outdoor access associated with increased fighting during the period of time when cats are being introduced (12), and a negative correlation between outdoor access and inter-cat affiliative behaviors (34). It is possible that cats with outdoor access may bring new and unfamiliar odors into the home which may initiate cat-cat conflict, however no research supports this. Another possibility is that cat owners may be more likely to let their cats outdoors when they do not get along in the home. Given our survey had a cross-sectional design, we were not able to assess temporality of factors associated with a negative cat-cat relationship, which induces uncertainty about causation (53). Future research should use a study design that allows for temporal investigation such as a longitudinal study where cats in the home can be followed over time. This type of study design would also help reduce recall bias which may exist in cross-sectional research.

A high proportion of participants reported that at least one of their cats have had, or currently has, a health problem (59.9%). Although this is concerning, it is comparable to other studies. For example, O'Neill et al. (54) found 68% (2,449/3,584) of cats seen by veterinarians in England have at least one health disorder. Moreover, 48% of US and Canadian cat owners (580/1,208) reported a health disorder in their own cat(s) (55). The most commonly reported health problems in the current study were obesity (25.2%) and dental disease (23.4%, Table 5) and other studies have reported similar prevalence of these health issues. Roberts and colleagues (17) found 19.9% (150/755) of UK owners reported their cats as overweight or obese. In the US, the prevalence of obesity in cats seen by veterinarians during 1995 (*N* = 8,159) was 35% (56), while more recently, Dodd and colleagues (55) found 33% (405/1,233) of US and Canadian cat owners rated their cat's body condition as overweight. Veterinary practices in the US (*N* = 15,226) report cat patients are most commonly diagnosed for dental calculus (24.2%) and gingivitis [13.1%; (56)]. Similarly, O'Neill et al. (54) report that periodontal disease was the most prevalent disease (13.9%, *N* = 499/3584) in cats of UK veterinary clinics. While the current study did not find any health issues to significantly impact the cat-cat relationship as perceived by the owner, future studies should assess if symptoms of health issues (i.e. pain, fatigue) would affect the latter.

A large portion of cat owners (78.5%) also reported that at least one of their cats has a current or previous behavior problem, with fears/phobias (45.7%), unwanted behaviors (45.2%) and destructive behaviors (40.6%) most commonly reported. However, we did not require these to be diagnosed by a veterinarian or animal behaviorist. One survey of US cat owners (*N* = 547), state that 47% of participants answered “yes” when asked if their cat(s) ever misbehave (9), and they found similar prevalence for anxiety/fear (59.4%) and destructive behaviors (49.7%) as the current study. Another survey of US and Canadian cat owners (*N* = 2465) found that 58% reported inappropriate scratching (57), which is similar in prevalence to destructive and unwanted behaviors in the current study. It is possible some participants selected a behavior problem because they have seen it in their cat(s) (i.e. excessive night time activity, unwanted behaviors, fear/phobias, etc.), but it may not be displayed to the intensity and frequency where it would constitute as a behavior problem (58).

Households with at least one cat that has shown animal or human-directed aggression were associated with owners rating their cats' relationship negatively. Aggression toward people and other animals can vary from subtle agonistic displays to more obvious displays that may lead to serious injuries. Inter-cat aggression is a major stressor for cats, and may lead to further behavioral problems such as house soiling (5, 6, 57), which may increase the risk of relinquishment (25–27). Aggression toward people and other animals may be affected by many factors such as socialization experiences, management of the home environment, and interactions with people and other animals in the home (58). It is important for the type of aggression to be identified (i.e., fear-related, territorial, play-related, petting-induced, redirected, social stress, pain-induced), as well as sources or triggers that may lead to an aggressive event (58). Research suggests cats reported to show human and/or animal-related aggression may involve redirected aggression, which is commonly enticed by inter-cat conflict and loud noises (59). However, the current study did not assess causes of aggression-related behavior problems given this is not possible with a cross-sectional survey design. Cat owners may benefit from addressing aggression through early management of the problem, to minimize the risk of stress, injuries, and further behavioral or health problems.

We also found that provision of resources in the home is associated with owner perceptions of a negative cat-cat relationship. Owners perceiving their cats' relationship as negative was associated with households with multiple litter boxes and feeding areas. These findings were not in line with our predictions; however, our survey design could not assess the temporality of these associations. Thus, it is possible that cat owners choose to provide multiple resources as a solution when conflict becomes present in the home rather than as a preventative measure. The American Association of Feline Practitioners recommends that multi-cat households should have multiple, easily accessible resources to meet cat behavior needs (60). Multi-cat households have been identified as a risk factor for behavior problems in the home (20, 22) such as inappropriate elimination (61–63), is a common reason for relinquishment (26). While providing multiple litter box areas may not solely prevent house soiling, it is an important consideration. In addition, providing multiple separate food areas in multi-cat households is recommended to help reduce agonistic interactions such as resource guarding, which may be present when one cat is more dominant and assertive over another more timid cat (60). Resource guarding around limited food areas may lead to rapid ingestion of food or inadequate nutritional intake, and may increase the risk of health issues overtime (64). Although the recommendation of multiple food areas is not based on scientific evidence, cats are naturally solitary hunters. Thus, feedings areas that are physically separate may reduce the potential of threat and may better mimic “solitary” eating (65, 66). Overall, there is little experimental evidence about the impact of resource distribution in the home on cat-cat interactions, and more research is needed.

Our survey results show that the cat-cat relationship in two-cat households is complex and impacted by many factors such as, cat sex, age, relatedness, outdoor access, resource provision in the home, and aggression directed toward other people and animals in the home. The complex interplay and directionality of these factors requires further investigation for a full understanding of how to improve the cat-cat relationship in the two-cat households. More research is also needed to provide cat owners with evidence-based recommendations for providing adequate resources in two-cat households. Cat owners may also benefit from information on factors to consider before acquiring a second cat to foster a stronger connection between their cats, as well as scientifically supported guidelines for introducing their cats.

### 4.1. Limitations

Our research survey was cross-sectional and limits our ability to understand the temporality of factors associated with participants' perceptions of their cats' relationship, thus limiting interpretations of the study results. In addition, the data may be impacted by participant recall bias and the responses received may be more indicative of their cats' current relationship. The results of this study are also reliant on the cat owner's ability to accurately assess their cats' relationship. The second part of the survey (results not published here) examines cat owner's knowledge of cat behavior and cat-cat interactions, and examines this in more detail.

The majority of the survey participants were female, middle aged (30–50 years old), had no dogs or children, and indicated they keep their cats indoor-only. A larger proportion of female participants is common in online survey studies (67) and a noted limitation. A large proportion of participants also had no children or dogs, which may be a limitation and reflection of the type of cat owner that participates in cat-related research surveys. Previous studies of cat owners also found the majority of participants do not have children (9, 18, 68, 69) and that approximately half (9) or the majority do not have dogs (18). Furthermore, it is possible our study attracted cat owners with a special interest in cat-related topics or research, which may not be representative of the average cat owner.

## Data availability statement

The raw data supporting the conclusions of this article will be made available by the authors, without undue reservation.

## Ethics statement

The studies involving human participants were reviewed and approved by UC Davis Institutional Research Ethics Board. Written informed consent for participation was not required for this study in accordance with the national legislation and the institutional requirements.

## Author contributions

SK wrote the manuscript. CM edited the manuscript. All authors established the study question, devised the study methods, and questionnaire. All authors contributed to the article and approved the submitted version.
