# Supplementary material for: Why can't we be friends? Exploring factors associated with cat owners' perceptions of the cat-cat relationship in two-cat households
Source: Front Vet Sci. 2023 Mar 27;10:1128757. doi: 10.3389/fvets.2023.1128757 (PMC10083434; doi:10.3389/fvets.2023.1128757)
Supplement: Supplementary file 1 [file Data_Sheet_1.docx]

**Supplementary File – Questionnaire**

**1. Inclusion Criteria:**

1. Are you at least 18 years of age?
2. Yes
3. No (skip to end of survey)
4. Where do you currently live?
5. Canada
6. USA
7. Other (skip to end of survey)
8. Are you the current primary owner of **two *companion cats** that spend at least 50 percent of their time indoors?

***Companion cats** live with a human owner and depend on them for their welfare.

1. Yes
2. No (skip to end of survey)

1. Are both of your cats approximately 1 year or older?
2. Yes
3. No (skip to end of survey)

**2. Owner Demographic Questions**

1. What is your current age in years?
2. 18-29
3. 30-39
4. 40-49
5. 50-59
6. 60-69
7. 70+
8. Prefer not to say, but over 18
9. What is your gender?
10. Male
11. Female
12. Non-binary
13. Other
14. Prefer not to say

*Displayed if “Where do you currently live” = USA*:

7. In which state do you currently reside?

▼ Alabama... Wyoming

*Displayed if “Where do you currently live” = Canada:*

1. In which province do you currently reside?

▼ Alberta... Yukon

1. Select the approximate **household area** that best describes your home.
2. Less than 500 square feet
3. 500-1000 square feet
4. 1000-1500 square feet
5. More than 1500 square feet
6. Prefer not to answer

1. How many dogs live in the same household as your cats?
2. 0
3. 1
4. 2
5. 3
6. 4+
7. Please state the number of adults, **including yourself**, and children that live in your household.

_____ **Adults** (18 years or older, including yourself)

_____ **Children** (Less than 18 years of age)

1. How **knowledgeable** are you about cat behavior?
2. Extremely
3. Very
4. Moderately
5. Somewhat
6. Not at all

1. Do you have any previous experience working with companion cats? (ex. animal hospital, grooming, training, daycares or pet-sitting, fostering, shelters)
2. Yes
3. No

*Displayed if “Do you have any previous experience working with companion cats?...” = Yes*:

1. Approximately, how many years of **combined experience** do you have working with companion cats?
2. Less than 1 year
3. 1-5
4. 6-10
5. 11-15
6. 16+

**3. Resource Provision Questions**

1. Are the **food bowls** provided for your cats: (Select all that apply)

▢ In the same room, side by side

▢ In the same room, not side by side

▢ In different rooms

▢ 1 or less food bowl provided

1. How many **scratching posts** are provided for your cats?

▼ 0 ... 10+

1. Are the **litter boxes** provided for your cats: (Select all that apply)

▢ In the same room, side by side

▢ In the same room, not side by side

▢ In different rooms

▢ 1 litter box is provided

▢ No litter box provided

1. Are the **sleeping areas** provided for your cats: (Select all that apply)

***Sleeping areas** can include cat beds, owners' bed, furniture, cat trees/hammocks.

▢ In the same room, side by side

▢ In the same room, not side by side

▢ In different rooms

▢ 1 sleeping area provided

▢ No sleeping area provided

**4. Cat Demographic Questions**

1. Enter the names of your cats below. The order does not matter.

First cat's name: _______

Second cat's name: _______

1. Select your cats' sex.

|  | Female Spayed | Male Neutered | Female Intact | Male Intact | Not Sure |
| --- | --- | --- | --- | --- | --- |
| {First Cat’s Name} | o | o | o | o | o |
| {Second Cat’s Name} | o | o | o | o | o |

1. Have your cats been ***declawed**?

***Declawed** cats do not have claws (or nails).

|  | No | Yes, all 4  paws | Yes, only front paws | Yes, only back paws | Not sure |
| --- | --- | --- | --- | --- | --- |
| {First Cat’s Name} | o | o | o | o | o |
| {Second Cat’s Name} | o | o | o | o | o |

1. Where did you obtain your cats from?

|  | Breeder | Pet store | Shelter or Rescue | Family, friend, relative, neighbor | Found as a stray or feral cat | Previous cat’s litter |
| --- | --- | --- | --- | --- | --- | --- |
| {First Cat’s Name} | o | o | o | o | o | o |
| {Second Cat’s Name} | o | o | o | o | o | o |

1. How old were your cats when you obtained them?

|  | **Kitten**  (Birth up to 1 year) | **Young Adult**  (1-3 years old) | **Adult**  (4-6 years old) | **Mature Adult**  (7-10 years  old) | **Senior**  (>10 years old) |
| --- | --- | --- | --- | --- | --- |
| {First Cat’s Name} | o | o | o | o | o |
| {Second Cat’s Name} | o | o | o | o | o |

1. What is the relationship between your cats?
   1. Not related
   2. Siblings
   3. Mother and daughter/son
   4. Father and daughter/son
   5. Other: ________
2. Select the **current approximate age** of each cat in years.

| {First Cat’s Name} | ▼ 1 ... 25 |
| --- | --- |
| {Second Cat’s Name} | ▼ 1 ... 25 |

1. Approximately, how long have your cats been living together?
   1. less than 1 year
   2. 1-3 years
   3. 4-6 years
   4. 7-9 years
   5. 10+ years

1. Select any current or previous health issue(s) diagnosed by a veterinarian for each cat. (Select all that apply)

|  | {First Cat’s Name} | {Second Cat’s Name} |
| --- | --- | --- |
| Diabetes mellitus | ▢ | ▢ |
| Dermatological disorders (ex. skin allergies) | ▢ | ▢ |
| Eye disorders (ex. conjunctivitis) | ▢ | ▢ |
| Gastrointestinal disorders (ex. diarrhea) | ▢ | ▢ |
| Heart disease | ▢ | ▢ |
| External parasites (ex. fleas, ticks, mites) | ▢ | ▢ |
| Hyperthyroidism | ▢ | ▢ |
| Non-obstructive urinary diseases | ▢ | ▢ |
| Internal parasites | ▢ | ▢ |
| Dental disease | ▢ | ▢ |
| Obesity | ▢ | ▢ |
| Obstructive urinary diseases | ▢ | ▢ |
| Osteoarthritis | ▢ | ▢ |
| Renal disease | ▢ | ▢ |
| Respiratory diseases | ▢ | ▢ |
| Other (please specify) | ▢ | ▢ |

1. Select any current or previous behavioral issue(s) for each cat. (Select all that apply)

|  | {First Cat’s Name} | {Second Cat’s Name} |
| --- | --- | --- |
| Aggression towards people | ▢ | ▢ |
| Aggression towards animals | ▢ | ▢ |
| Destructive behaviors (ex. scratching) | ▢ | ▢ |
| GI and ingestive disorders (ex. pica, coprophagia) | ▢ | ▢ |
| Excessive night time activity | ▢ | ▢ |
| Fear/phobias (ex. noise phobias) | ▢ | ▢ |
| Separation anxiety | ▢ | ▢ |
| Unwanted behaviors (ex.  jumping on counters, stealing food) | ▢ | ▢ |
| Stereotypic and compulsive disorders (ex. chasing tails, excessive licking) | ▢ | ▢ |
| Other (please specify) | ▢ | ▢ |

1. How would you describe your cats' first encounter?
   1. Extremely positive
   2. Somewhat positive
   3. Neutral
   4. Somewhat negative
   5. Extremely negative
   6. Previously introduced
   7. Not sure
2. Overall, how would you rate the relationship between your cats?
   1. Extremely negative
   2. Somewhat negative
   3. Neither positive nor negative
   4. Somewhat positive
   5. Extremely positive
3. Select {First Cat’s Name}'s breed.
   1. Domestic (not purebred)
   2. Purebred
   3. Purebred Mix
   4. Not sure
4. Select **all the colors** that closely resemble {First Cat’s Name} 's coat color? (Picture examples provided)

▢ Beige

▢ Black

▢ Brown

▢ Grey

▢ Lavender

▢ Orange

▢ Red

▢ White

1. Which of the following best describes {First Cat’s Name}'s coat pattern? (Select all that apply). (Picture examples provided)

▢ **Solid:** One color (Example: all white, all grey, all black, etc.)

▢ **Bi-color:** Solid white with another solid color

▢ **Calico:** Patches of white, black and orange

▢ **Tabby:** Including classic, mackerel, spotted, and ticked. Vertical bands, spirals, or spots across body.

▢ **Tortoiseshell:** Solid black or chocolate with orange patches

▢ **Other** (please describe the coat pattern): _______________

1. Which best describes {First Cat’s Name}'s outdoor access?
2. Strictly indoor
3. Indoor with supervised outdoor access (eg. unleashed supervision, leashed cats, and cat patios)
4. Indoor with unsupervised outdoor access
5. Select {Second Cat’s Name}'s breed.
6. Domestic (not purebred)
7. Purebred
8. Purebred Mix
9. Not sure
10. Select **all the colors** that closely resemble {Second Cat’s Name} 's coat color? (Picture examples provided)

▢ Beige

▢ Black

▢ Brown

▢ Grey

▢ Lavender

▢ Orange

▢ Red

▢ White

1. Which of the following best describes {Second Cat’s Name}'s coat pattern? (Select all that apply). (Picture examples provided)

▢ **Solid:** One color (Example: all white, all grey, all black, etc.)

▢ **Bi-color:** Solid white with another solid color

▢ **Calico:** Patches of white, black and orange

▢ **Tabby:** Including classic, mackerel, spotted, and ticked. Vertical bands, spirals, or spots across body.

▢ **Tortoiseshell:** Solid black or chocolate with orange patches

▢ **Other** (please describe the coat pattern): _______________

1. Which best describes {Second Cat’s Name}'s outdoor access?
2. Strictly indoor
3. Indoor with supervised outdoor access (eg. unleashed supervision, leashed cats, and
4. cat patios)
5. Indoor with unsupervised outdoor access

**5. Video Questions**

You will be shown 10 short (up to 10-seconds) videos **without sound.**

Please **watch the videos carefully** as you will be asked questions about each video. Any vocalizations from the cats will be displayed using subtitles.


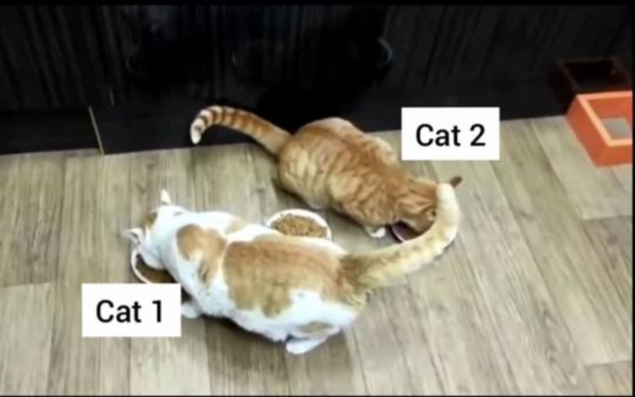


1. How would you describe the **overall** interaction between the cats in the video?
   1. Extremely negative
   2. Somewhat negative
   3. Neither positive nor negative
   4. Somewhat positive
   5. Extremely positive
   6. Not sure
2. How would you rate **Cat 1's** experience in the video?
3. Extremely negative
4. Somewhat negative
5. Neither positive nor negative
6. Somewhat positive
7. Extremely positive
8. Not sure
9. How would you rate **Cat 2's** experience in the video?
10. Extremely negative
11. Somewhat negative
12. Neither positive nor negative
13. Somewhat positive
14. Extremely positive
15. Not sure
16. Having seen the video, how often do **your own cats** display similar behaviors?
17. Never
18. Rarely
19. Sometimes
20. Often
21. Always
22. Not sure


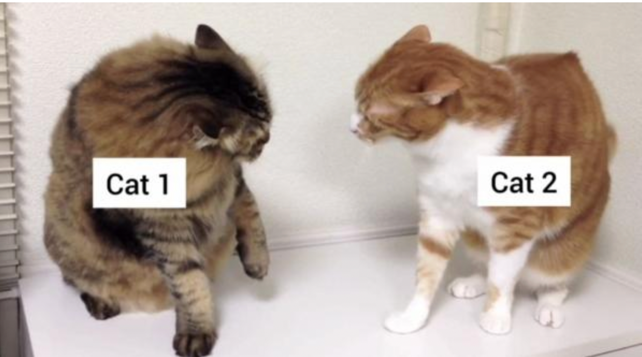


1. How would you describe the **overall** interaction between the cats in the video?
   1. Extremely positive
   2. Somewhat positive
   3. Neither positive nor negative
   4. Somewhat negative
   5. Extremely negative
   6. Not sure
2. How would you rate **Cat 1's** experience in the video?
3. Extremely positive
4. Somewhat positive
5. Neither positive nor negative
6. Somewhat negative
7. Extremely negative
8. Not sure

1. How would you rate **Cat 2's** experience in the video?
2. Extremely positive
3. Somewhat positive
4. Neither positive nor negative
5. Somewhat negative
6. Extremely negative
7. Not sure
8. Having seen the video, how often do **your own cats** display similar behaviors?
9. Never
10. Rarely
11. Sometimes
12. Often
13. Always
14. Not sure


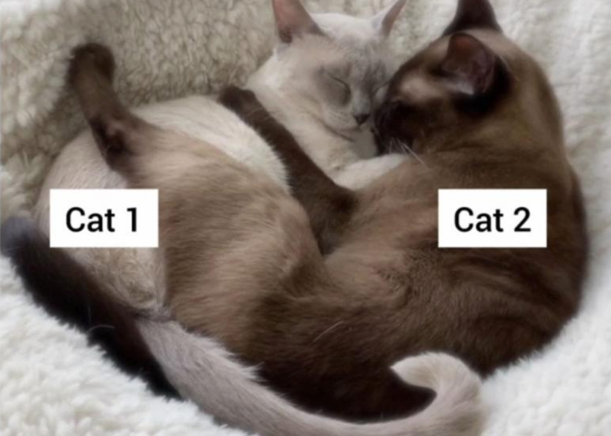


1. How would you describe the **overall** interaction between the cats in the video?
   1. Extremely positive
   2. Somewhat positive
   3. Neither positive nor negative
   4. Somewhat negative
   5. Extremely negative
   6. Not sure
2. How would you rate **Cat 1's** experience in the video?
3. Extremely positive
4. Somewhat positive
5. Neither positive nor negative
6. Somewhat negative
7. Extremely negative
8. Not sure

1. How would you rate **Cat 2's** experience in the video?
2. Extremely positive
3. Somewhat positive
4. Neither positive nor negative
5. Somewhat negative
6. Extremely negative
7. Not sure
8. Having seen the video, how often do **your own cats** display similar behaviors?
9. Never
10. Rarely
11. Sometimes
12. Often
13. Always
14. Not sure


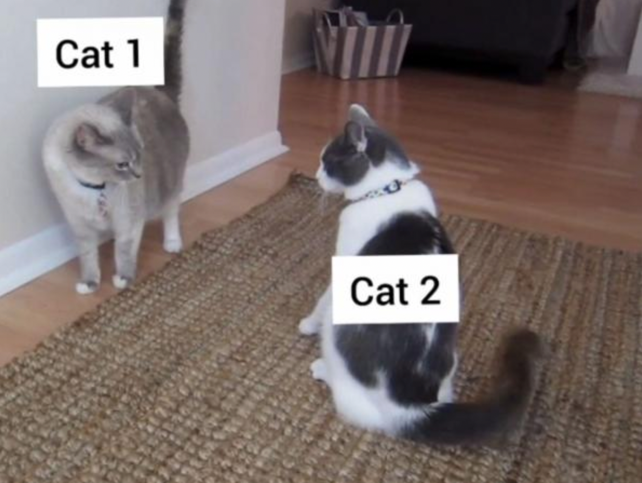


1. How would you describe the **overall** interaction between the cats in the video?
   1. Extremely negative
   2. Somewhat negative
   3. Neither positive nor negative
   4. Somewhat positive
   5. Extremely positive
   6. Not sure
2. How would you rate **Cat 1's** experience in the video?
3. Extremely negative
4. Somewhat negative
5. Neither positive nor negative
6. Somewhat positive
7. Extremely positive
8. Not sure
9. How would you rate **Cat 2's** experience in the video?
10. Extremely negative
11. Somewhat negative
12. Neither positive nor negative
13. Somewhat positive
14. Extremely positive
15. Not sure
16. Having seen the video, how often do **your own cats** display similar behaviors?
17. Never
18. Rarely
19. Sometimes
20. Often
21. Always
22. Not sure


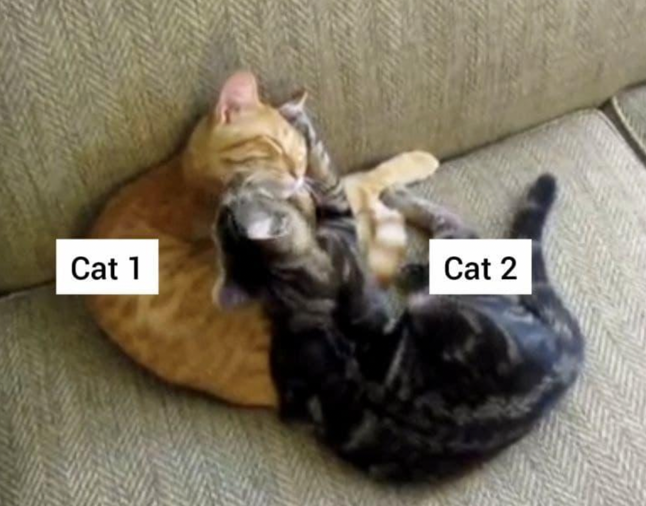


1. How would you describe the **overall** interaction between the cats in the video?
   1. Extremely positive
   2. Somewhat positive
   3. Neither positive nor negative
   4. Somewhat negative
   5. Extremely negative
   6. Not sure
2. How would you rate **Cat 1's** experience in the video?
3. Extremely positive
4. Somewhat positive
5. Neither positive nor negative
6. Somewhat negative
7. Extremely negative
8. Not sure

1. How would you rate **Cat 2's** experience in the video?
2. Extremely positive
3. Somewhat positive
4. Neither positive nor negative
5. Somewhat negative
6. Extremely negative
7. Not sure
8. Having seen the video, how often do **your own cats** display similar behaviors?
9. Never
10. Rarely
11. Sometimes
12. Often
13. Always
14. Not sure


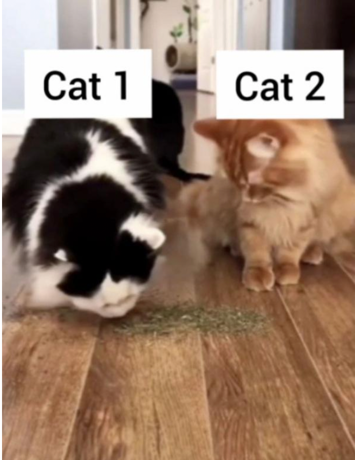


1. How would you describe the **overall** interaction between the cats in the video?
   1. Extremely negative
   2. Somewhat negative
   3. Neither positive nor negative
   4. Somewhat positive
   5. Extremely positive
   6. Not sure
2. How would you rate **Cat 1's** experience in the video?
3. Extremely negative
4. Somewhat negative
5. Neither positive nor negative
6. Somewhat positive
7. Extremely positive
8. Not sure
9. How would you rate **Cat 2's** experience in the video?
10. Extremely negative
11. Somewhat negative
12. Neither positive nor negative
13. Somewhat positive
14. Extremely positive
15. Not sure
16. Having seen the video, how often do **your own cats** display similar behaviors?
17. Never
18. Rarely
19. Sometimes
20. Often
21. Always
22. Not sure


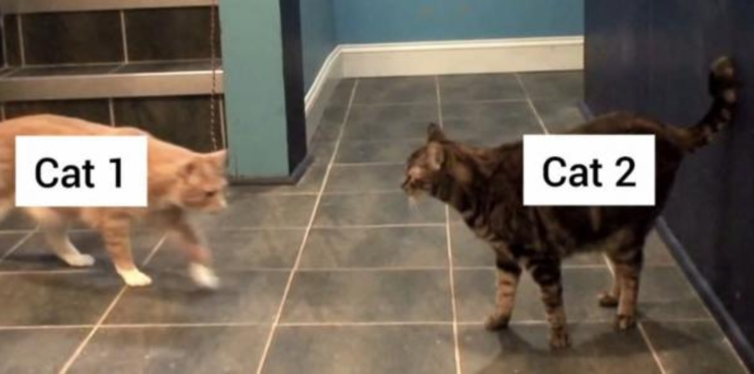


1. How would you describe the **overall** interaction between the cats in the video?
   1. Extremely negative
   2. Somewhat negative
   3. Neither positive nor negative
   4. Somewhat positive
   5. Extremely positive
   6. Not sure
2. How would you rate **Cat 1's** experience in the video?
3. Extremely negative
4. Somewhat negative
5. Neither positive nor negative
6. Somewhat positive
7. Extremely positive
8. Not sure
9. How would you rate **Cat 2's** experience in the video?
10. Extremely negative
11. Somewhat negative
12. Neither positive nor negative
13. Somewhat positive
14. Extremely positive
15. Not sure
16. Having seen the video, how often do **your own cats** display similar behaviors?
17. Never
18. Rarely
19. Sometimes
20. Often
21. Always
22. Not sure


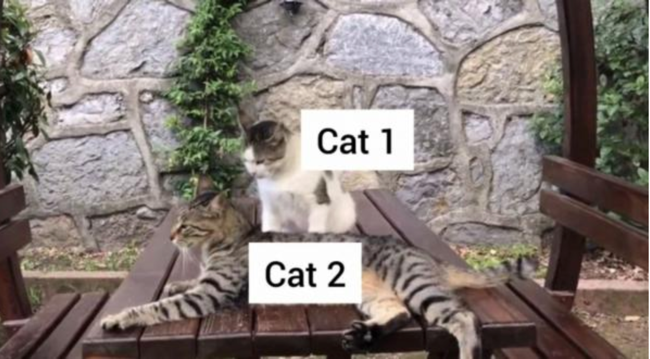


1. How would you describe the **overall** interaction between the cats in the video?
   1. Extremely positive
   2. Somewhat positive
   3. Neither positive nor negative
   4. Somewhat negative
   5. Extremely negative
   6. Not sure
2. How would you rate **Cat 1's** experience in the video?
3. Extremely positive
4. Somewhat positive
5. Neither positive nor negative
6. Somewhat negative
7. Extremely negative
8. Not sure

1. How would you rate **Cat 2's** experience in the video?
2. Extremely positive
3. Somewhat positive
4. Neither positive nor negative
5. Somewhat negative
6. Extremely negative
7. Not sure
8. Having seen the video, how often do **your own cats** display similar behaviors?
9. Never
10. Rarely
11. Sometimes
12. Often
13. Always
14. Not sure


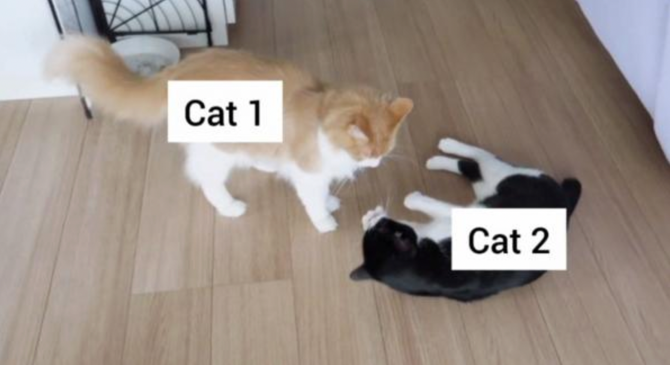


1. How would you describe the **overall** interaction between the cats in the video?
   1. Extremely negative
   2. Somewhat negative
   3. Neither positive nor negative
   4. Somewhat positive
   5. Extremely positive
   6. Not sure
2. How would you rate **Cat 1's** experience in the video?
3. Extremely negative
4. Somewhat negative
5. Neither positive nor negative
6. Somewhat positive
7. Extremely positive
8. Not sure
9. How would you rate **Cat 2's** experience in the video?
10. Extremely negative
11. Somewhat negative
12. Neither positive nor negative
13. Somewhat positive
14. Extremely positive
15. Not sure
16. Having seen the video, how often do **your own cats** display similar behaviors?
17. Never
18. Rarely
19. Sometimes
20. Often
21. Always
22. Not sure


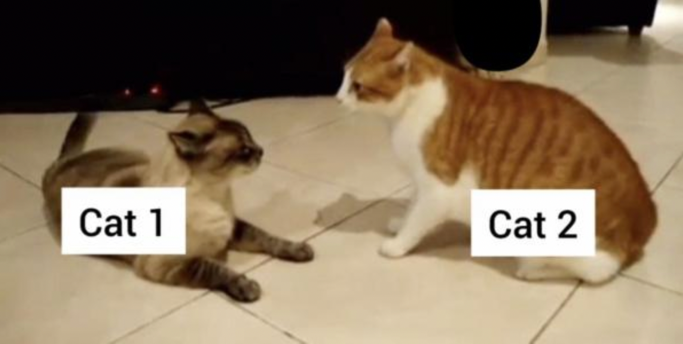


1. How would you describe the **overall** interaction between the cats in the video?
2. Extremely positive
3. Somewhat positive
4. Neither positive nor negative
5. Somewhat negative
6. Extremely negative
7. Not sure
8. How would you rate **Cat 1's** experience in the video?
9. Extremely positive
10. Somewhat positive
11. Neither positive nor negative
12. Somewhat negative
13. Extremely negative
14. Not sure

1. How would you rate **Cat 2's** experience in the video?
2. Extremely positive
3. Somewhat positive
4. Neither positive nor negative
5. Somewhat negative
6. Extremely negative
7. Not sure
8. Having seen the video, how often do **your own cats** display similar behaviors?
9. Never
10. Rarely
11. Sometimes
12. Often
13. Always
14. Not sure
